# Network analysis of rehabilitation needs and influencing factors in aged patients recovering from first-time stroke

**DOI:** 10.3389/fneur.2026.1817787

**Published:** 2026-05-15

**Authors:** Yumeng Gong, Jiajia Chen, Dongyu Hu, Xiumu Yang, Juanjuan Wang, Yongli Duan

**Affiliations:** 1 School of Nursing, Bengbu Medical University, Bengbu, Anhui, China; 2 Fuyang People's Hospital, Fuyang, Anhui, China

**Keywords:** aged, first-ever stroke, influencing factors, network analysis, recovery period of cerebral infarction, rehabilitation needs

## Abstract

**Objective:**

To explore the rehabilitation needs and influencing factors in aged patients recovering from cerebral infarction.

**Methods:**

A cross-sectional survey was conducted from July to December 2025, selecting 436 aged patients recovering from their first cerebral infarction in the neurology department of a top-tier hospital in Anhui Province. The survey included general information, and scales for the following: stroke rehabilitation needs, the Barthel Index (ADL scale), social support, and self-efficacy.

**Results:**

The average rehabilitation needs score was 140.60 ± 29.806. The key rehabilitation needs identified were assistance with putting on and taking off clothes and shoes (rs = 1.256), swallowing function training (rs = 1.221), support for feelings of inferiority (rs = 1.208), personal hygiene help (rs = 1.200), and understanding of the discharge follow-up plan (rs = 1.197). Network analysis showed that self-care ability, social support, and self-efficacy significantly impacted rehabilitation needs.

**Discussion:**

In older adults recovering from their first-ever ischemic stroke, rehabilitation needs are closely connected. The central need is assistance with dressing, including putting on and taking off clothes and shoes. These needs are shaped by multiple factors, such as lesion location on the right side, early participation in rehabilitation, self-care ability, self-efficacy, and social support, all of which play key roles. Therefore, rehabilitation interventions should consider both patients' specific needs and the factors that influence them. Tailored, precise care can better address their rehabilitation needs and enhance their quality of life.

## 1 Introduction

Stroke is a leading cause of disability worldwide, with ischemic stroke being the most common. Its high incidence, disability rates, and recurrence have become major public health issues (1). As the population ages, the number of aged patients with ischemic stroke in China continues to rise (2). In clinical practice, aged patients in the recovery phase after their first-ever ischemic stroke often face significant challenges when adapting to rehabilitation (3), principally due to a lack of experience. These challenges include functional impairments after the acute phase, insufficient understanding of the disease, stress from psychological adjustments, and a lack of rehabilitation information and support systems (4–6). Traditional research has examined the factors affecting a single rehabilitation need using linear models; however, this approach does not capture the complex interactions and network characteristics of various factors (7). On the contrary, network analysis builds connection networks between variables, making it easier to visualize how different factors interact and can also help identify key nodes and important connections in the system, which can provide a theoretical basis for more targeted and multidimensional rehabilitation interventions (8). In this study, we applied network analysis to develop a model of the factors influencing the rehabilitation needs of ischemic stroke patients during recovery, exploring how these needs interact and identifying the core factors that influence recovery. Our goal is to offer empirical insights to better meet patients' rehabilitation needs.

## 2 Materials and method

### 2.1 Survey subjects

Using a cross-sectional survey, the study selected patients who were hospitalized in the neurology department of a third-level Class-A hospital in Anhui Province from July 2025 to December 2025. These patients were in the recovery phase after a stroke.

Patients who met the following criteria were included: 1. The diagnostic criteria of the “Chinese Guidelines for Acute Ischemic Stroke Diagnosis and Treatment 2023” (9); 2. 60 years or older, experiencing their first-ever stroke; 3. With stable vital signs, who were in the recovery phase; and 4. Who could communicate normally with the surveyor.

The following patients were excluded: 1. Presenting with severe dysfunction in important organs such as the heart, liver, or kidneys; 2. Diagnosed with neurodegenerative diseases, such as dementia, Parkinson's disease, or motor neuron disease; and 3. History of other serious neurological diseases, such as myasthenia gravis, multiple sclerosis, or spinal cord injury.

Based on relevant research from network analysis (10), this study included 28 nodes. The sample size was 5–10 times the number of variables, accounting for a 10% inefficiency rate. Therefore, the sample size should be between 154 and 308 cases. According to the requirements for mixed network sample size (11), the sample size must exceed the total number of parameters (including the threshold and pairwise association parameters). The threshold parameters should be equal to the number of nodes (N), and the formula for pairwise association parameters should be N × (N−1)/2. In this study, the network consisted of ten nodes (five dimensions of rehabilitation needs and five influencing factors). Therefore, the minimum sample size needed was 10 × (10−1)/2 = 45. The final sample size in this study was 436, sufficient to ensure adequate statistical power for the network analysis and approved by the hospital's ethics committee (FK20245554-2).

### 2.2 Survey tools

#### 2.2.1 General information survey

Based on a literature review, we created a survey that included questions about gender, age, occupation, marital status, education level, sources of income, income situation, types of insurance, housing situation, primary caregiver, and whether there is impaired limb movement (assessed from medical records, with muscle strength score < 4 indicating impairment) (12), affected body parts, whether thrombolysis treatment was received, the number of chronic diseases, and rehabilitation exercise status.

#### 2.2.2 Stroke rehabilitation needs scale

The Stroke Rehabilitation Needs Scale was developed by Zhao et al. (13), and is used to assess the rehabilitation needs of stroke patients in China. It consists of 28 items across five dimensions, with a total score ranging from 28 to 140. Each item is rated using a 5-point Likert scale, where 1 to 5 represents “not important” to “very important.” The physiological needs dimension includes six items, the psychological needs (five items), the self-esteem/respect needs (three items), the information needs (eight items), and the rehabilitation needs (six items). The total score for each dimension is calculated by dividing the sum of its items by the number of items. The scale demonstrated acceptable reliability, with a Cronbach's α coefficient of 0.731.

#### 2.2.3 Barthel index (BI)

The Barthel Index (BI) was designed by Mahoney and Barthel in the mid-1950s to assess a person's ability to perform daily activities (14). The scale includes 10 items, and the score ranges from 0 to 100. A score of 100 indicates normal functioning, between 61 and 99 is mild, 41 and 60 is moderate, 20 and 40 is severe, and 0 and 19 means complete disability. The Cronbach's alpha coefficient for this scale is 0.89.

#### 2.2.4 General self-efficacy scale (GSES)

This scale was developed by Schwarzer et al. (15) and later translated into Chinese by Zhang et al. (16). It is a one-dimensional scale with 10 items. Participants rate each item using a 4-point Likert scale, ranging from [1] for “Strongly Disagree” to [4] for “Strongly Agree.” The total score is obtained by adding the scores for all 10 items and dividing the sum by 10. An average score of 2.5 serves as the midpoint, with scores above 2.5 indicating a high level of self-efficacy and scores below 2.5 indicating a lower level of self-efficacy. The scale has a Cronbach's alpha of 0.870.

#### 2.2.5 Perceived social support scale (PSSS)

This study used the Chinese version of the PSSS created by Blumenthal et al. (17) and revised by Jiang Qianjin (18). The scale consists of three dimensions: family (four items), friend (four items), and other (four items) supports, with a total of 12 items rated on a 7-point scale (1–7 points). The total score ranges from 12 to 84 points, with higher scores indicating greater social support for the patient. The Cronbach's alpha coefficient of the scale is 0.942.

### 2.3 Data collection and quality control

Before the survey, two researchers were trained to ensure familiarity with the assessment tools and their proper use. The discharge date was set as the survey event, given the patient's recovery. During the research, the team was to carefully follow the inclusion and exclusion criteria when selecting participants. After explaining the research goals and obtaining informed consent, the researchers conducted one-on-one interviews with the patients. Once the survey is completed, the team checked the data on-site for completeness, accuracy, and authenticity. All questionnaires were reviewed and entered into the system by two people.

### 2.4 Statistical analysis

We performed descriptive analyses using SPSS 27.0. Categorical data are shown as counts and percentages. Rehabilitation need scores did not follow a normal distribution, so we reported them as medians with interquartile ranges (M(P25, P75)), and provided means (x ± s) for reference. Spearman's correlation was used to examine relationships between variables, with correlation coefficients (*r*) indicating the strength of linear associations. Variables with *P* < 0.05 in univariate analysis were included in a multivariate logistic regression model to assess their independent effects on outcomes. We built a Gaussian graphical model (GGM) in R 4.4.2 to map the network of rehabilitation needs. Connections between rehabilitation needs and influencing factors were modeled using a mixed graphical model (MGM) (19). The network's edges show how well one node predicts another, and larger edge values indicate stronger associations. Red edges represent positive, and blue dashed edges indicate negative correlations. We calculated centrality measures—strength (rs), closeness (rc), and betweenness (rb)—to evaluate node importance. Nodes with higher strength represent core symptoms. Betweenness reflects a node's role as a bridge connecting other nodes, and closeness shows its overall centrality in the network. Sentinel symptoms occur frequently but have low centrality. While connecting to a few nodes, these nodes are highly influential. Node stability was assessed using the correlation stability (CS) coefficient, which should be at least 0.25 and ideally above 0.5 (20).

## 3 Results

### 3.1 General information of stroke recovery patients

A total of 440 surveys were distributed, and 436 valid responses were received, with a response rate of 99.09%. General information is shown in Table 1.

**Table 1:** General information of first-ever subacute stroke patients and univariate analysis results of rehabilitation needs (*n* = 436).

Item	Frequency (percentage %)	Rehabilitation requirement (score)		Statistical measurement	*P*
		*M*(*P*_25_ and *P*_75_)	Mean		
Gender					
Male	263 (60.32%)	133.00 (117.00, 163.00)	139.24 ± 29.75	−1.238^1)^	0.216
Female	173 (39.68%)	139.00 (120.50, 165.50)	142.65 ± 29.86		
Age (years)					
60 ~ 75 years	274 (62.84%)	132.00 (116.00, 152.25)	135.53 ± 28.46	−4.519^1)^	0.001
≥75 years	162 (37.16%)	147.00 (125.50, 174.00)	149.16 ± 30.17		
Education level					
Below middle school	353 (80.96%)	137.00 (120.00, 164.00)	141.70 ± 29.87	3.299^2)^	0.192
High school or vocational school	57 (13.07%)	132.00 (113.50, 161.00)	136.96 ± 31.91		
University or above	26 (5.96%)	129.50 (116.25, 144.25)	133.54 ± 22.68		
Marital status					
Married	418 (95.87%)	136.00 (118.00, 163.25)	140.50 ± 29.80	−0.494^1)^	0.621
Unmarried/divorced/widowed	18 (4.13%)	142.00 (122.00, 170.25)	142.72 ± 30.83		
Pre-retirement employment					
Steady job	111 (25.46%)	132.00 (115.00, 157.00)	137.27 ± 30.11	−1.622^1)^	0.105
Unemployed	325 (74.54%)	138.00 (120.00, 164.50)	141.73 ± 29.66		
Living arrangement					
Lives alone	13 (2.98%)	138.00 (123.50, 164.00)	137.54 ± 27.88	−0.026^1)^	0.979
Does not live alone	423 (97.02%)	136.00 (118.00, 164.00)	140.69 ± 29.89		
Main caregiver					
Spouse	123 (28.21%)	126.00 (112.00, 151.00)	132.79 ± 26.13	16.680^2)^	0.001
Adult children	302 (69.27%)	139.00 (122.00, 169.00)	144.37 ± 30.49		
No caregiver	11 (2.52%)	128.00 (92.00, 142.00)	124.27 ± 29.23		
Main source of income					
Pension	99 (22.71%)	133.00 (115.00, 163.00)	137.78 ± 29.60	2.063^2)^	0.356
Family support	312 (71.56%)	137.00 (118.25, 164.00)	141.14 ± 29.90		
Minimum living allowance	25 (5.73%)	143.00 (126.50, 165.50)	144.96 ± 29.74		
Health insurance type					
Employee health insurance	98 (22.48%)	132.00 (114.75, 158.50)	137.44 ± 29.78	2.623^2)^	0.269
NRCMS	335 (76.83%)	137.00 (119.00, 164.00)	141.42 ± 29.89		
Self-pay	3 (0.69%)	155.00 (133.00, ——-)	151.33 ± 16.80		
Monthly income					
< 3000	337 (77.29%)	137.00 (119.00, 164.50)	141.45 ± 29.97	−1.241^1)^	0.215
≥3000	99 (22.71%)	133.00 (116.00, 157.00)	137.71 ± 29.22		
Affected body parts					
Left side	169 (38.76%)	142.00 (123.50, 169.00)	145.66 ± 30.15	8.371^2)^	0.015
Right side	152 (34.86%)	132.00 (116.25, 155.v75v	137.23 ± 29.35		
Both sides	115 (26.38%)	133.00 (116.00, 161.00v	137.61 ± 29.12		
Presence of mobility limitations					
Yes	376 (86.24%)	137.50 (122.00, 166.00)	142.86 ± 29.65	−4.082^1)^	0.001
No	60 (13.76%)	120.50 (108.00, 142.25)	126.43 ± 26.97		
		*M*(*P*_25_ **and** *P*_75_**)**	**Mean**		
Engagement in rehabilitation training					
Yes	105 (24.08%)	156.00 (129.50, 176.00)	154.85 ± 30.16	−5.287^1)^	0.001
No	331 (75.92%)	133.00 (116.00, 154.00)	136.08 ± 28.27		
Number of chronic diseases					
0	23 (5.28%)	136.00 (117.00, 152.00)	137.48 ± 31.25	3.539^2)^	0.316
1	92 (21.10%)	131.50 (117.00, 165.50)	140.24 ± 30.36		
3	161 (36.93%)	133.00 (116.00, 156.00)	138.48 ± 30.15		
4	160 (36.70%)	139.00 (122.00, 166.00)	143.48 ± 28.98		
Acute-phase reperfusion therapy					
Yes	60 (13.76%)	130.50 (112.50, 143.00)	133.83 ± 25.81	−1.913^1)^	0.056
No	376 (86.24%)	137.00 (118.00, 165.75)	141.68 ± 30.29		

^1)^z-value; ^2)^H-value.

### 3.2 Network analysis of rehabilitation needs in the recovery phase of aged patients with first-time cerebral infarction

**Figure 1:**
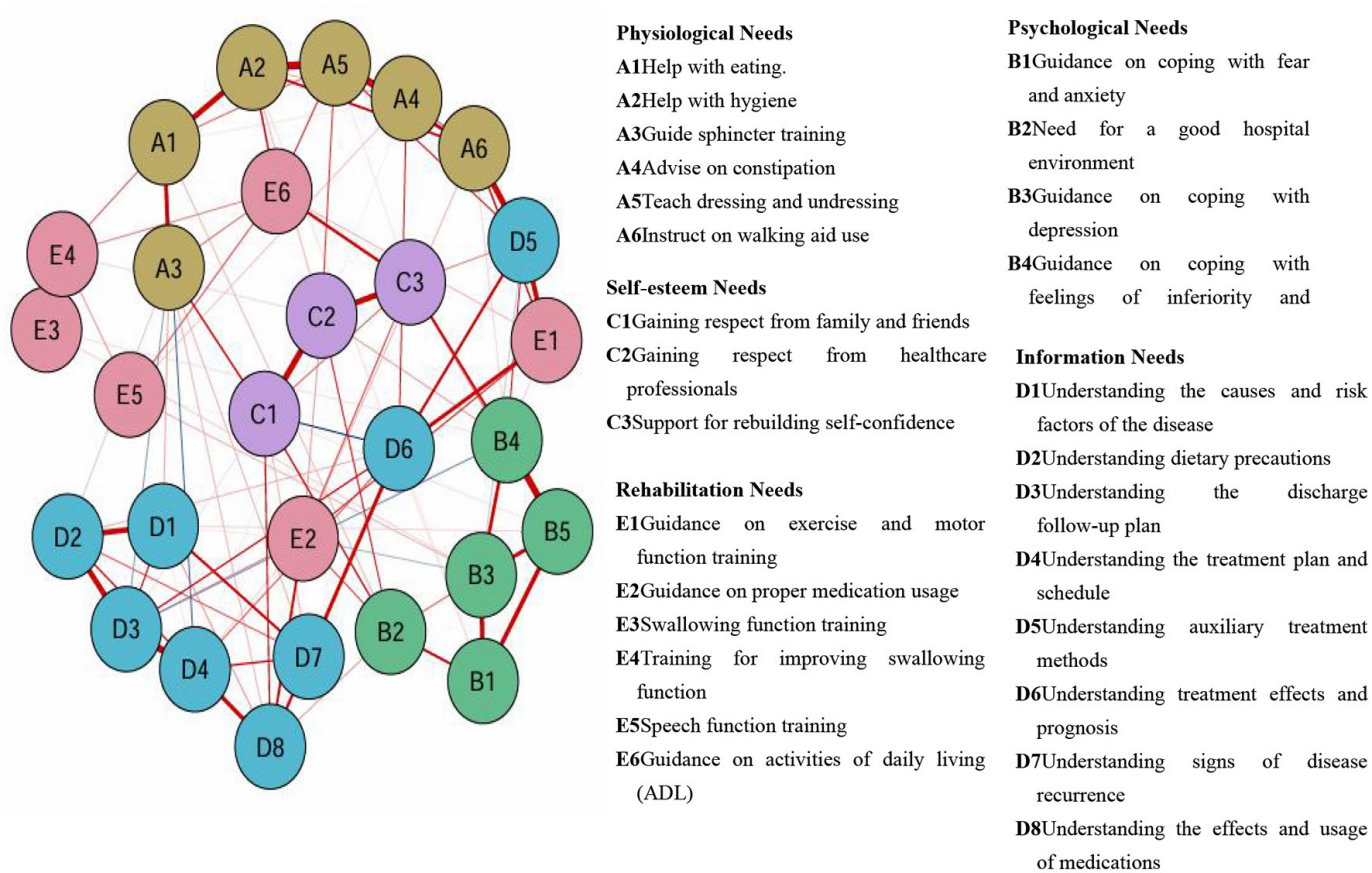
Symptom network of rehabilitation needs in aged patients with first-ever cerebral infarction during the recovery phase (*n* = 436).

The results showed that most rehabilitation needs were positively correlated, indicating their interconnection. Centrality analysis revealed that the top five rehabilitation needs that ranked by intensity were assistance with dressing and undressing, including putting on shoes and socks (rs = 1.256), training to improve swallowing function (rs = 1.221), guidance on coping with feelings of inferiority and uselessness (rs = 1.208), help with personal hygiene (e.g., washing, grooming, and brushing teeth) (rs = 1.200), and understanding the discharge follow-up plan (rs = 1.197). The top five rehabilitation needs that ranked by closeness centrality were gaining respect from healthcare professionals (rc = 0.00245), from family (spouse and relatives) and friends (rc = 0.00246), helping to rebuild self-confidence (rc = 0.00238), understanding treatment effects and prognosis (rc = 0.00234), and learning about available auxiliary treatment options (e.g., massage and use of support; rc = 0.00241) (see Figure 1). In this study, the correlation stability coefficients for intensity, closeness centrality, and intermediary centrality were 0.75, 0.672, and 0.206, respectively, indicating that the intensity and closeness centrality results were stable. Intensity was chosen as the main criterion for determining core rehabilitation needs.

### 3.3 Correlation between BI, social support, and self-efficacy scores in aged patients during the recovery phase of first-time cerebral infarction, and their relationship with rehabilitation needs scores

Spearman's correlation analysis showed that, in older patients during the recovery phase of the first ischemic stroke, the BI, social support, and self-efficacy scores were negatively correlated with rehabilitation needs (refer to Table 2).

**Table 2:** Correlations among activities of daily living (BI), perceived social support, self-efficacy, and rehabilitation needs in aged patients with first-ever cerebral infarction during the recovery phase **(*n* =** **436)**.

Item	Score		Rehabilitation needs	
	*M* (*P*_25_ and *P*_75_)	Mean	*R*	*P*
Barthel index	90.00 (71.25, 90.00)	81.230 ± 15.820	−0.651	0.001
Social support score	49.00 (42.00, 58.00)	50.280 ± 10.405	−0.356	0.001
Self-efficacy score	1.70 (1.40, 2.20)	1.814 ± 0.539	−0.550	0.001

### 3.4 Multivariate analysis of rehabilitation needs in aged patients with acute ischemic stroke during the recovery phase

The rehabilitation needs score was used as the dependent variable. Variables that were statistically significant in the univariate analysis were selected as independent variables for multiple linear regression. The values assigned to the independent variables are shown in Table 3.

**Table 3:** Assignment methods for independent variables.

Independent variable	Assignment method
Age (years)	60 < 75years = 1; ≥75years = 2
Main caregiver	Spouse = 1; (Z_1_ = 0, Z_2_ = 0) Children = 2; (Z_1_ = 1, Z_2_ = 0) No caregiver = 3. (Z_1_ = 0, Z_2_ = 1)
Affected body part	Left side (Z_1_ = 0, Z_2_ = 0) Right side (Z_1_ = 1, Z_2_ = 0); Both sides (Z_1_ = 0, Z_2_ = 1)
Presence of mobility limitations	Yes = 1; No = 2
Engagement in rehabilitation training	Yes = 1; No = 2
Barthel index (ADI)	Raw data entry
Social support scale	Raw data entry
Self-efficacy level	Raw data entry

Regression analysis showed that lesion location (right side), participation in rehabilitation training, BI, social support, and self-efficacy were factors influencing rehabilitation needs in older patients during the recovery phase of the first ischemic stroke. All independent variables together explained 51.2% of the variance in rehabilitation needs scores and no multicollinearity was observed among the variables (refer to Table 4).

**Table 4:** Linear regression analysis of rehabilitation needs in first-episode aged patients with stroke in the recovery period.

Item	Regression coefficient	Standard error	Standardized coefficient	*t*	*P*	VIF
Constant	271.485	9.233	–	29.404	0.001	–
Affected body part (right side)	−7.192	2.346	−0.155	−3.066	0.002	1.256
Rehabilitation training	−5.679	2.525	−0.082	−2.250	0.025	1.171
Barthel index (ADL)	−0.909	0.077	−0.482	−11.819	0.001	1.484
Social Support Scale	−0.375	0.119	−0.131	−3.154	0.002	1.538
Self-efficacy Level	−11.994	2.427	−0.217	−4.942	0.001	1.716

R2 = 0.523, Adjusted R2 = 0.512, F = 46.598, *P* < 0.001.

### 3.5 Mixed network analysis of rehabilitation needs in aged patients with acute stroke during recovery phase

**Figure 2:**
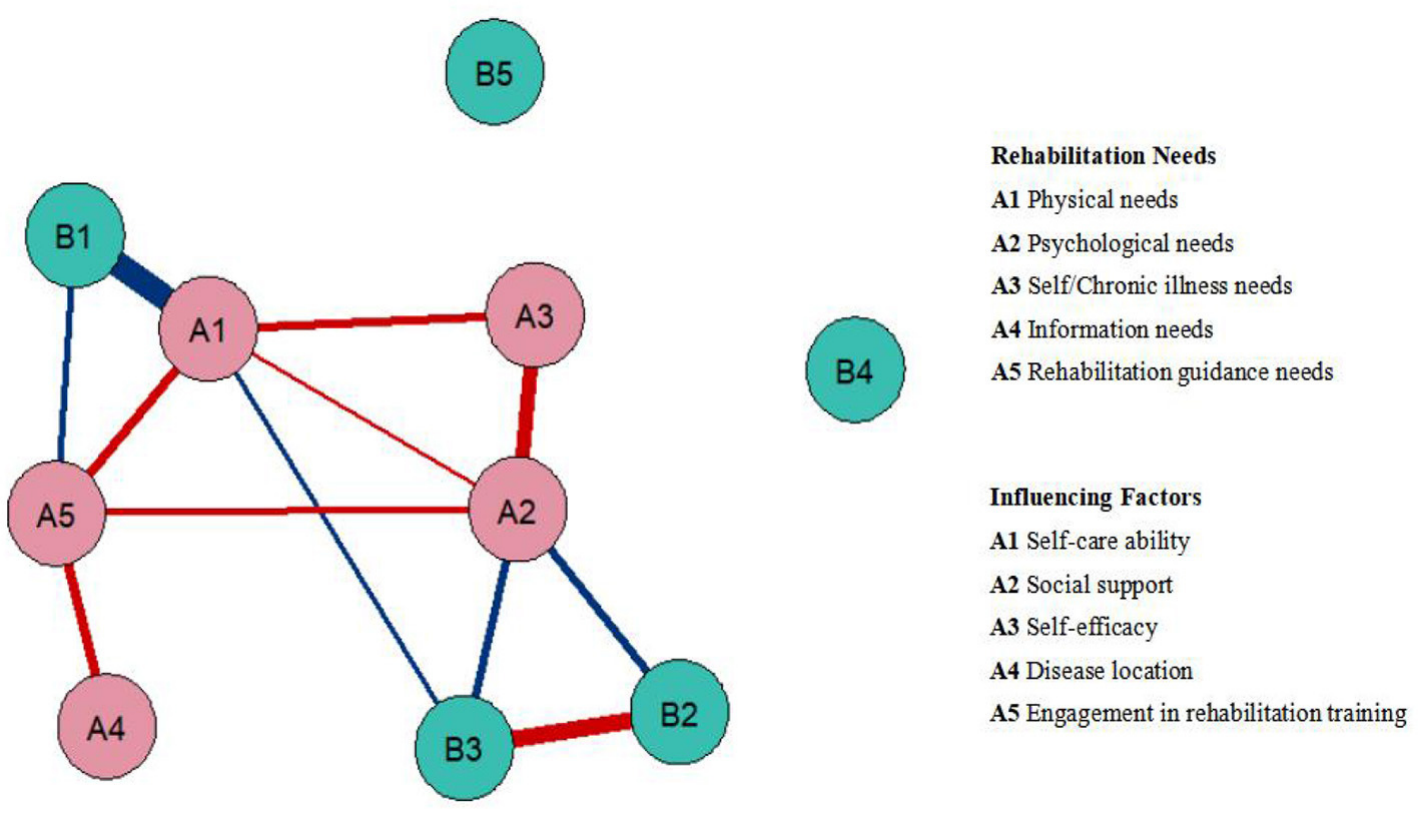
Network diagram of rehabilitation needs and their associated factors in aged patients with first-ever cerebral infarction during the recovery phase.

The results showed the following: B2 social support was negatively correlated with A2 psychological needs (edge = −0.162); B1 self-care ability was negatively correlated with A1 physical needs (edge = −0.519) and A5 rehabilitation guidance needs (edge = −0.123); B3 self-efficacy was negatively correlated with A1 physical needs (edge = −0.094) and A2 psychological needs (edge = −0.150). A2 psychological needs were positively correlated with A1 physical needs (edge = 0.052), A3 self-esteem needs (edge = 0.312), and A5 rehabilitation guidance needs (edge = 0.111). A1 physical needs were positively correlated with A3 self-esteem needs (edge = 0.179) and A5 rehabilitation guidance needs (edge = 0.208). The A4 information needs were positively correlated with the A5 rehabilitation guidance needs (edge = 0.201). B2 social support positively correlated with B3 self-efficacy (edge = 0.337; see Figure 2).

The node centrality indicators showed that the most influential factor was B1's self-care ability (rs = 0.642). The CS coefficient of the model was 0.75, indicating the good stability of the network model.

## 4 Discussion

### 4.1 Identification of core needs in the rehabilitation demand network

The results of this study showed that helping patients put on and take off socks, improving swallowing function, guiding them to cope with feelings of low self-esteem and uselessness, assisting with personal hygiene, and providing information about follow-up after discharge occupy central positions in the rehabilitation needs network. Tasks such as dressing and personal hygiene involve fine motor skills, coordination, balance, and joint movement. Hand function is difficult to restore, and patients often have lasting impairments, which can seriously limit their ability to take care of themselves and lead to feelings of low self-worth and uselessness (21). Studies have shown that integrating functional training into real-life situations can effectively improve patients' abilities (22). Early rehabilitation should include joint exercises, finger grip, and upper limb coordination training. Family members should be trained in proper assistance techniques, and communication should be encouraged to avoid over-helping. Patients can then practice dressing, washing, and toileting in daily-life scenarios using aids such as dressing sticks, pull rings, Velcro-fastened clothing, and non-slip mats. Later, home self-care training should be strengthened. Group rehabilitation and psychological interventions can also help reduce negative emotions.

Our study highlights that improving swallowing disorders is a common complication in older adults after cerebral infarction, affecting approximately 40%−60% of patients during the acute phase and increasing the risk of pneumonia, with mortality rates reaching 10.45% (23–25). Liqun et al. (6) noted that the rehabilitation needs of patients with swallowing difficulties persist throughout the disease course, and recent evidence shows that multidirectional resistance jaw-forward exercises combined with bundled care strategies can effectively enhance swallowing function, improve nutritional and biochemical markers, and reduce the risk of aspiration (26, 27). Moving forward, integrating individualized assessment, neuromodulation techniques, and intelligent rehabilitation technologies could enable more comprehensive interventions and better address patients' needs, thereby improving swallowing function.

Additionally, follow-up after discharge is a core rehabilitation requirement during the recovery period (28). One study (29) implemented a hospital–community integrated smart stroke follow-up platform. Patient records were transferred to the community 24 h before discharge, and community nurses conducted regular follow-ups, monitored the data, and uploaded it in real time. Any changes in the patient's condition prompted a referral back to the hospital, enabling coordinated management among the hospital, the community, and the patient. This system effectively supported neurological recovery, improved patients' daily self-care and self-management skills, and helped control vascular risk factors. Moving forward, integrating personalized rehabilitation plans with smart follow-up platforms and wearable devices could provide continuous monitoring and guidance throughout recovery, enhancing patient adherence and improving rehabilitation outcomes after discharge.

In summary, dressing and undressing, swallowing training, coping with feelings of inferiority and uselessness, performing personal hygiene, and understanding the discharge follow-up plan are the core within the rehabilitation needs network. Based on the International Classification of Functioning, Disability and Health (ICF) framework, these needs correspond to body functions (swallowing training), activities (dressing and personal hygiene), participation (rebuilding confidence and self-esteem), and environmental factors (follow-up information), reflecting the multidimensional rehabilitation needs of aged patients with ischemic stroke. They represent key components of individual rehabilitation. Clinically, multidisciplinary collaboration should be employed using functional training, activity-based practice, psychological support, and improved follow-up plans to deliver systematic, precise rehabilitation interventions that address patients' rehabilitation needs more effectively.

### 4.2 Analysis of factors influencing rehabilitation needs

Linear regression analysis showed that lesion location (right side), participation in rehabilitation training, self-care ability, social support, and self-efficacy were factors influencing rehabilitation needs in older patients during the recovery period after their first ischemic stroke. Mixed network analysis found that lesion location and rehabilitation training were not associated with specific dimensions of rehabilitation needs, suggesting that these factors may primarily affect the overall level of patient needs. This may be because lesion location and rehabilitation training have a comprehensive impact on post-stroke recovery. Studies have shown that lesion location is associated with functional and cognitive outcomes in stroke patients and that rehabilitation training can improve multiple outcomes, including activities of daily living, motor function, balance, and gait. This indicates that lesion location and rehabilitation training may influence the overall level of rehabilitation needs through the combined effects of multiple factors, rather than affecting only a single specific dimension (30–32).

Mixed network analysis showed that higher levels of social support and self-efficacy were associated with lower psychological needs; patients with low self-care ability and low self-efficacy tended to have higher physiological needs. Support from family, friends, and healthcare providers can enhance patients' sense of control over their illness and confidence in rehabilitation, thereby improving self-efficacy, buffering negative emotions such as anxiety, inferiority, and helplessness, and influencing physiological rehabilitation needs while promoting functional recovery (33, 34). Huaxue et al. (33) found that cognitively engaging family support effectively improved stroke patients' self-efficacy and rehabilitation outcomes. Wan et al. (35) reported that nurse-led peer support can enhance patients' social participation and self-efficacy, reduce psychological distress, and improve quality of life. Future interventions should focus on increasing social support and strengthening self-efficacy to reduce psychological problems and rehabilitation needs, enhance the effectiveness of rehabilitation guidance, and promote the recovery of physiological functions.

## 5 Conclusion

This study used network analysis to identify the core needs of aged patients in the recovery phase of a first stroke. The main needs were assistance with dressing, undressing, and putting on shoes and socks. The study also found that self-care ability, social support, and self-efficacy are key factors that affect rehabilitation needs. This suggests that healthcare professionals should identify the most pressing needs and focus on interventions that enhance patients' self-efficacy and strengthen their social support networks.

### 5.1 Limitations

This study used a single-center, cross-sectional design, which limits the ability to determine causal relationships among rehabilitation needs. Caution should be taken when generalizing the results to other regions or populations. Future studies could use longitudinal designs to verify causal relationships among different rehabilitation needs and develop nursing interventions based on core needs, which could then be tested in clinical practice. In addition, detailed information on the content, intensity, and standardization of patient rehabilitation was lacking, limiting the interpretability of the results. Future research could consider using subgroup analysis or propensity score-adjusted models to control for potential confounding factors and more clearly clarify the relationships among different rehabilitation needs.

## Data Availability

The original contributions presented in the study are included in the article/supplementary material, further inquiries can be directed to the corresponding author.
